# Treatment of Tuberculosis in a Region with High Drug Resistance: Outcomes, Drug Resistance Amplification and Re-Infection

**DOI:** 10.1371/journal.pone.0023081

**Published:** 2011-08-23

**Authors:** Maryline Bonnet, Manuela Pardini, Francesca Meacci, Germano Orrù, Hasan Yesilkaya, Thierry Jarosz, Peter W. Andrew, Mike Barer, Francesco Checchi, Heinz Rinder, Graziella Orefici, Sabine Rüsch-Gerdes, Lanfranco Fattorini, Marco Rinaldo Oggioni, Juliet Melzer, Stefan Niemann, Francis Varaine

**Affiliations:** 1 Clinical Research department, Epicentre, Geneva, Switzerland; 2 Dipartimento di Malattie Infettive Parassitarie e Immunomediate, Istituto Superiore di Sanità, Rome, Italy; 3 Dipartimento di Biologia Molecolare, Università di Siena, Siena, Italy; 4 Dipartimento di Scienze Odontostomatologiche, Università di Cagliari, Cagliari, Italy; 5 Department of Infection, Immunity and Inflammation, University of Leicester, Leicester, United Kingdom; 6 Essai clinique Evaluation Epidemiologie Statistiques, Paris, France; 7 Sachgebiet Parasitologie Bayerisches, Landesamt für Gesundheit und Lebensmittelsicherheit, Oberschleißheim, Germany; 8 National Reference Center for Mycobacteriology, Forschungszentrum Borstel, Borstel, Germany; 9 Medical department, Médecins Sans Frontières, Paris, France; University of Stellenbosch, South Africa

## Abstract

**Introduction:**

Emerging antituberculosis drug resistance is a serious threat for tuberculosis (TB) control, especially in Eastern European countries.

**Methods:**

We combined drug susceptibility results and molecular strain typing data with treatment outcome reports to assess the influence of drug resistance on TB treatment outcomes in a prospective cohort of patients from Abkhazia (Georgia). Patients received individualized treatment regimens based on drug susceptibility testing (DST) results. Definitions for antituberculosis drug resistance and treatment outcomes were in line with current WHO recommendations. First and second line DST, and molecular typing were performed in a supranational laboratory for *Mycobacterium tuberculosis* (MTB) strains from consecutive sputum smear-positive TB patients at baseline and during treatment.

**Results:**

At baseline, *MTB* strains were fully drug-susceptible in 189/326 (58.0%) of patients. Resistance to at least H or R (PDR-TB) and multidrug-resistance (MDR-TB) were found in 69/326 (21.2%) and 68/326 (20.9%) of strains, respectively. Three MDR-TB strains were also extensively resistant (XDR-TB). During treatment, 3/189 (1.6%) fully susceptible patients at baseline were re-infected with a MDR-TB strain and 2/58 (3.4%) PDR-TB patients became MDR-TB due to resistance amplification. 5/47 (10.6%) MDR- patients became XDR-TB during treatment. Treatment success was observed in 161/189 (85.2%), 54/69 (78.3%) and 22/68 (32.3%) of patients with fully drug susceptible, PDR- and MDR-TB, respectively. Development of ofloxacin resistance was significantly associated with a negative treatment outcome.

**Conclusion:**

In Abkhazia, a region with high prevalence of drug resistant TB, the use of individualized MDR-TB treatment regimens resulted in poor treatment outcomes and XDR-TB amplification. Nosocomial transmission of MDR-TB emphasizes the importance of infection control in hospitals.

## Introduction

Antituberculosis drug resistance is a serious threat to the achievement of the goal of the Stop TB partnership to eliminate tuberculosis (TB) as a public health problem by 2050 [1]. Multidrug-resistance (MDR-TB) is defined as resistance to isoniazid (H) and rifampicin (R). Extensive drug-resistance (XDR-TB) is defined as MDR-TB plus resistance to a fluoroquinolone and any one of the second-line injectable drugs (capreomycin, amikacin or kanamycin). The World Health Organization (WHO), estimated a total number of 440,000 cases and 150,000 deaths due to MDR-TB in the year 2008 [2]. A recent investigation showed that up to 15% of MDR-TB strains worldwide are already XDR-TB [3].

Current guidelines for drug resistant TB management are based on expert opinion and case series. Furthermore, there are very few reports on treatment outcomes of regimens for mono- or poly-drug resistant TB. Overall, MDR-TB is associated with much poorer treatment outcomes compared with drug susceptible TB [4]–[8]. These poor outcomes are linked with longer periods of infectivity, which result in enhanced transmission of drug resistant *Mycobacterium tuberculosis* (*MTB*) strains [9].

Treatment of MDR-TB patients appears to entail a considerable risk of creating XDR-TB with the potential of direct transmission of XDR-TB strains [10]. Reasons for resistance amplification in MDR-TB cases on individualised second line treatment regimen urgently require further systematic investigation [11].

*MTB* drug resistance is particularly prevalent in Eastern part of Europe or in Central Asia, where 12 countries have reported proportions of MDR-TB rates of 6% or more among new TB cases [2]. In such scenarios of highly prevalent *MTB* drug resistance, current recommendations include systematically performing drug susceptibility testing (DST) at the time of TB diagnosis, initiating standardized short course chemotherapy until DST results are available and adapting treatment in case drug resistance is found [12]. This treatment strategy was introduced in 2001 by Médecins Sans Frontières (MSF) in Abkhazia, an autonomous region of western Georgia with an estimated population of 150,000 inhabitants. The TB incidence was 84/100,000 inhabitants in 2008 and more than 8% of new cases were identified as MDR-TB [13], [14]. Approximately 20% of *MTB* strains harbour the specific phylogenetic lineage Beijing genotype that was identified as an independent risk factor of MDR-TB and *MTB* transmission, indicating a potential role of strains with Beijing genotype in the epidemiology of the drug resistant TB (DR-TB) in the region [15], [16].

We performed an in depth investigation of the effectiveness of the TB treatment strategy in Abkhazia. Working within an existing prospective cohort, we combined DST results, molecular strain typing data and reports of treatment outcomes to identify predictors of negative treatment outcomes and to describe DR-TB amplification and re-infection during treatment.

## Methods

### Study design and population

Following informed consent, all patients aged ≥18 years old with sputum smear-positive pulmonary TB, presenting to the Gulripsch TB referral centre in Abkhazia (Georgia), were enrolled between March 2003 and September 2005 in a prospective cohort study. Due to the chronic conflict situation with Georgia since 1994, the Abkhazia TB control program has limited connection with the national TB program of Georgia. The program has received support from Médecins Sans Frontières for diagnosis and treatment of all TB cases since 1999.

### Treatment strategy and patient follow-up

TB screening was performed based on clinical symptoms, chest X-ray and sputum smear microscopy examination. TB case definitions followed WHO/IUATLD guidelines [12], [17]. All patients were started on empirical standard short course chemotherapy with H, R, pyrazinamide (Z) and ethambutol (E). Streptomycin (S) was added for patients with a previous TB treatment history. Treatment was then adapted according to DST results, as described in Table 1. Prior to 2^nd^ line drug DST results, MDR-TB patients were given an empiric MDR regimen (see Table 1). This regimen was further adapted, according to 2^nd^ line drugs DST results, in order to achieve a minimum of six months intensive phase containing at least four 2^nd^ line drugs to which the *MTB* strain was highly likely susceptible. Clofazimine and amoxicillin-clavulanate were added to the regimen when the number of highly likely effective 2^nd^ line drugs was insufficient. The first line drugs, E and Z were continued if found susceptible. The intensive phase lasted a minimum of 6 months and at least 4 months after culture conversion. All non MDR-TB patients presenting with a *MTB* resistance to at least H or R were given an adapted treatment regimen (Table 1). Though this group included mono- and poly-drug resistant TB, for the sake of conciseness, these patients were named as PDR-TB.

**Table 1:** Treatment according to drug resistance patterns.

Resistance	Intensive phase	Continuation phase
S, E or ES	2 H R Z E	4 H R
MDR*	≥6 Cm-Ofx/Mx-Eto/Pto PAS Cs	18 Ofx/Mx Eto/Pto PAS Cs
H or HS	2H R Z E	7 R E Z
HE or HES	3 Cm/Km Ofx/Mx R Z	7 Ofx/Mx R Z
R or RS	3 Cm/Km Ofx/Mx E Z	12 Ofx/Mx E Z
RE or RES	3 Cm/Km Ofx/Mx H Z	12 Ofx/Mx H Z

E: ethambutol; S: streptomycin; H: isoniazid; MDR: multidrug resistance; Cm: capreomycin; Km: kanamycin; Ofx: ofloxacin; Mx: moxifloxacin; Eto: ethionamide; Pto: prothionamide; PAS: paramino salicylic acid; Cs: cycloserine.

*Empiric treatment further adapted to DST to 2nd line drugs.

The continuation phase lasted between seven and twelve months for PDR-TB and eighteen months for MDR-TB, following completion of the intensive phase, provided patients remained continuously smear- and culture-negative throughout this period. Patients' response was monitored by monthly sputum smear and culture examination. Culture was not repeated in PDR-TB patients with smear-negative follow-up results and good clinical response. After two months with positive culture results, PDR-TB patients received an empirical MDR-TB regimen (Cm, FQ, Eto, PAS and Cs) whilst waiting for DST results. Patients were hospitalized during the entire intensive phase and had monthly outpatient visits during the continuation phase. Blood count, liver, and renal function tests and serum electrolytes were performed regularly. Early and aggressive management of side effects was provided. Treatment was delivered free of charge and under direct observation and with strong psycho-social support during the entire course of treatment. The WHO Green Light Committee approved the program in 2004.

### Laboratory procedures

Smear microscopy used the hot Ziehl Neelsen method. Two sputum specimens of all smear-positive patients were sent to the supra-national TB laboratory at the Istituto Superiore di Sanità, Roma (Italy) for culture and DST. Specimens were processed by the N-acetyl-L-cysteine-NaOH (NALC) method and inoculated into Lowenstein-Jensen (LJ) medium (Biomérieux, Marcy l'Etoile, France) and BACTEC MGIT 960 (MGIT) tubes (Becton-Dickinson), according to manufacturer's instructions. LJ slants were incubated at 37°C in 5% CO_2_ and controlled weekly for 8 weeks. TB strains were identified in positive cultures by DNA probes (Gene Probe, San Diego, Ca). DST for H, R, E and S was carried out using the MGIT system. In case of resistance to 1^st^ line drugs, DST for Ofx, Km, Cm, Eto, PAS and Cs was performed using the proportion methods on 7H10, as previously described [15]. Isolated strains were shipped to the Research Centre Borstel (Germany) for molecular typing. Extraction of genomic DNA from mycobacterial strains and DNA fingerprinting, using IS*6110* as a probe, were performed according to a standardized protocol [18]. Additionally, all isolates were analyzed by the spoligotyping technique [19]. Molecular typing data were analyzed with Bionumerics software (version 4·5; Applied Maths, Sint-Martens-Latem, Belgium). Spoligotyping data were used to confirm strain relationships and for genotype classification according to SpolDB4 and the MIRU-VNTRplus webpage [19]–[21]. In addition, we screened IS*6110* Restriction Fragment Length Polymorphism (RFLP) and spoligotyping patterns for mixed infections (infection with two strains).

### Definitions of treatment outcome, drug resistance amplification and re-infection

WHO definitions of treatment outcome definitions were used for patients susceptible to first line drugs or resistant to S, E or ES [12], [17]. For MDR-TB, a patient was defined as “cured” if s/he completed the treatment and had at least five consecutive negative culture results during the final twelve months of treatment. If only one positive culture was reported during that time without concomitant clinical evidence of deterioration, the patient was still considered as being cured, provided that this positive culture was followed by a minimum of three consecutive negative cultures, recorded at least 30 days apart. Patient was declared as “failure” if two or more of the five cultures recorded in the final twelve months were positive, or if any one of the final three cultures was positive, or if a clinical decision was made to discontinue treatment early due to poor response or adverse events. For PDR-TB, patient was defined as “cured” if s/he completed treatment and had at least three consecutive negative smear/culture results during the final six months of treatment. Patient was declared as “failure” if having at least one positive culture after three months of adapted regimen.

For both MDR- and PDR-TB, “treatment completion” was defined as a patient who completed treatment but did not meet the definition for “cure” due to lack of bacteriological results. “Death” was defined by the occurrence of death during treatment regardless of the cause and “defaulting” if the patient missed at least two consecutive months of treatment. The combination of “cure” and “treatment completed” defined “success”. Culture conversion was reported using the latest WHO case definition of two consecutive negative smears and cultures taken 30 days apart [12].

Drug resistance amplification was defined as an increase in the number of drugs towards which *MTB* was resistant *in vitro* during treatment follow-up compared with baseline, if both baseline and follow-up strains were genetically identical. If strains were different, the increase in drug resistance was attributed to a re-infection.

### Statistical analysis

Clinical and laboratory data were entered into a database using SQLServer (Microsoft Visual Studio.NET 7·0) and analyzed in StataSE™, 9^th^ version, College Station, TX. Treatment outcomes were presented according to patients' baseline DST patterns. For MDR-TB patients, treatment outcomes were also presented according to any baseline resistance to second line drugs. The proportion of culture converted patients who reverted to culture positivity was calculated. When tabulating proportions of patients who became resistant during treatment, patients without complete follow-up laboratory information were excluded from the analysis. To calculate the drug resistance amplification and exogenous re-infection patients with mixed or double infection were excluded. Univariate analysis was performed to identify factors associated with a negative outcome of MDR-TB treatment (death or failure) among baseline patients' characteristics (age, gender, TB treatment history and prisoner history) and biological markers (DST results and molecular findings), after exclusion of defaulters. Because there were very few missing observations, they were excluded from the analysis.

The study was approved by the health authorities of Abkhazia and the Ministry of Health of Georgia.

## Results

A total of 405 smear-positive pulmonary TB patients were diagnosed in the Gulripsch hospital between March 2003 and September 2005. Out of these, 366 (90.4%) met the inclusion criteria. Data from 326 patients were included in the final analysis. The 40 exclusions were: due either to shipment problems with sputum specimen for *MTB* culture for 11 cases, 3 cases with culture contamination and 26 cases with baseline negative culture results.

Table 2 shows patients' baseline characteristics. A total of 195 patients (59.8%) had strains resistant to at least one 1^st^ line drug and 135 (41.4%) patients were at least resistant to isoniazid. Among the 195 patients, 57 (29.3%) had strains resistant to at least one 2^nd^ line drug. A total of 69 patients were PDR-TB (21.2%) and 68 were MDR-TB (20.9%). Among PDR-TB, 2 (2.9%) were resistance to R. Among MDR-TB cases, 3 were XDR-TB (4.5%). The median number of drugs (1^st^ and 2^nd^ line), to which MDR-TB patients were resistant to was 4.5 drugs, interquartile range (IQR) [4]–[6]. One fourth harboured a strain belonging to the Beijing family.

**Table 2:** Baseline characteristics of patients, N = 326.

	Results
Sex ratio Male: Female	252∶74
Age, mean (Standard Deviation)	42 (14)
Past TB treatment history n (%)	127 (39.1)^a^
Former prisoner, n (%)	38 (11.7)^b^
Baseline drug resistance pattern, n (%)	
Fully susceptible or resistant to S, E or SE	189 (58.0)
Monoresistance to H	27 (8.3)
Resistance to HS	29 (8.9)
Resistance to EH +-S	11 (3.4)
Monoresistance to R+-S	2 (0.6)
HR+-S resistance	48 (14.7)
HRE+-S resistance	20 (6.1)
Among MDR, n (%)	68 (20.9)^c^
Resistance to at least one 2^nd^ line drug	36 (52.9)
Resistance to ethionamide	25 (37.3)
Resistance to ofloxacin	3 (4.5)
Resistance to kanamycin and/or capreomycin	21 (31.4)
XDR	3 (4.5)
Infection with Beijing strain^d^, n (%) N = 311	78 (25.1)

a 1 missing information.

b 2 missing informations.

c One case without DST result for all 2^nd^ line drugs.

d Exclusion of 15 patients with mixed or double infection.

E: ethambutol; S: streptomycin; H: isoniazid; MDR: multidrug resistance; XDR: extensively drug-resistance.

The overall treatment success rate was 72.7% (237/326), defaulting rate 16.2% (53/326), death rate 7.7% (25/326), and failure rate 3.4% (11/326). Table 3 shows treatment outcomes according to drug resistance patterns. Success rate was 85.2% for patients with fully drug susceptible patients, and 78.3% for patients with PDR-TB strains. There was no statistically significant difference in success rate between patients with full susceptible, H+-S, or HE+-S resistant strains (p = 0.67). The small number of PDR-TB resistant to R didn't allow comparison of outcome with other PDR-TB.

**Table 3:** Tuberculosis treatment outcomes according to baseline drug resistance patterns.

	Full susceptible and resistant to E, S or ES n (%)	PDR*		MDR n (%)
		H or HS resistance n (%)	HE or HES resistance n (%)	
N	189	56	11	68
Positive outcome (success)	161(85.2)	45 (80.3)	9 (81.8)	22 (32.3)
Cured	154 (81.5)	42 (75)	6 (54.5)	13 (19.1)
Completed	7 (3.7)	3 (5.4)	3 (27.3)	9 (13.2)
Negative outcome	11 (5.8)	3 (5.4)	1 (9.1)	22 (32.3)
Failure	4 (2.1)	2 (3.6)	0	5 (7.3)
Death	7 (3.7)	1 (1.8)	1 (9.1)	16 (23.5)
Defaulter	17 (9)	8 (14.3)	1 (9.1)	25 (36.7)

* Two patients were also resistant to rifampicin and rifampicin + streptomycin. Both were defaulters.

E: ethambutol; S: streptomycin; H: isoniazid; PDR: polydrug resistance; MDR: multidrug resistance.

Almost one fourth of MDR-TB patients died after a median period of treatment of 1 month, IQR [0–7.6]. One third discontinued treatment after a median period of 6.6 months IQR [4–12.9].

MDR-TB treatment success rate was 41.9% (13/31) for patients without resistance to 2^nd^ line drugs and 27.8% (10/36) for patients with resistance to any 2^nd^ line drug. However, this difference was non-significant (p = 0.22). Out of the 3 XDR-TB patients 2 died and 1 defaulted.

Out of 68 MDR-TB patients, 37 (54.4%) converted to culture negative. After exclusion of 21 patients who died or defaulted during the first 6 months of treatment, the culture conversion rate was 78.7% (37/47). Median time to culture conversion was 6 months IQR [4]–[7]. Among the 37 patients who converted, 4 (10.8%) reverted to culture positive during treatment at 3 (n = 2), 6 (n = 1) and 8 (n = 1) months after culture conversion. Of these 4 patients, 1 failed treatment, 2 died and 1 defaulted.

None of the baseline MDR-TB patients' characteristics were significantly associated with a negative outcome (Table 4).

**Table 4:** Predictors of poor treatment outcomes of MDR-TB patients: univariate analysis (N = 43).

Characteristics		n	Negative outcomes %	P-value
Gender	Male	5	52.6	0.20
	Female	38	20.0	
Age group	≤30	7	42.9	0.10
	30–39	16	37.5	
	40–49	15	73.3	
	50 and more	5	20.0	
Patient's type*	Previously Treated Cases	31	51.6	0.38
	New Cases	11	36.4	
Prisoner history	Yes	35	62.5	0.46
	No	8	45.7	
Beijing strain**	Yes	30	35.7	0.27
	No	9	53.6	
Baseline 2^nd^ line resistance	Yes	21	52.4	0.76
	No	22	45.5	
Baseline resistance to Ofx*	Yes	2	100	0.22
	No	40	45	
Baseline resistance to Km*	Yes	11	54.5	0.73
	No	31	45.2	
Number of drugs resistant to	<4 drugs	9	33.3	0.75
	4 drugs	14	57.1	
	5 drugs	11	45.4	
	≥6 drugs	9	55.6	
Increase of Ofx resistance*		Yes	100	<0.001
		No	18.5	

*Missing information.

**After exclusion of 4 cases with mixed or double infection.

Km: kanamycin; Ofx: ofloxacin.

**Figure 1:**
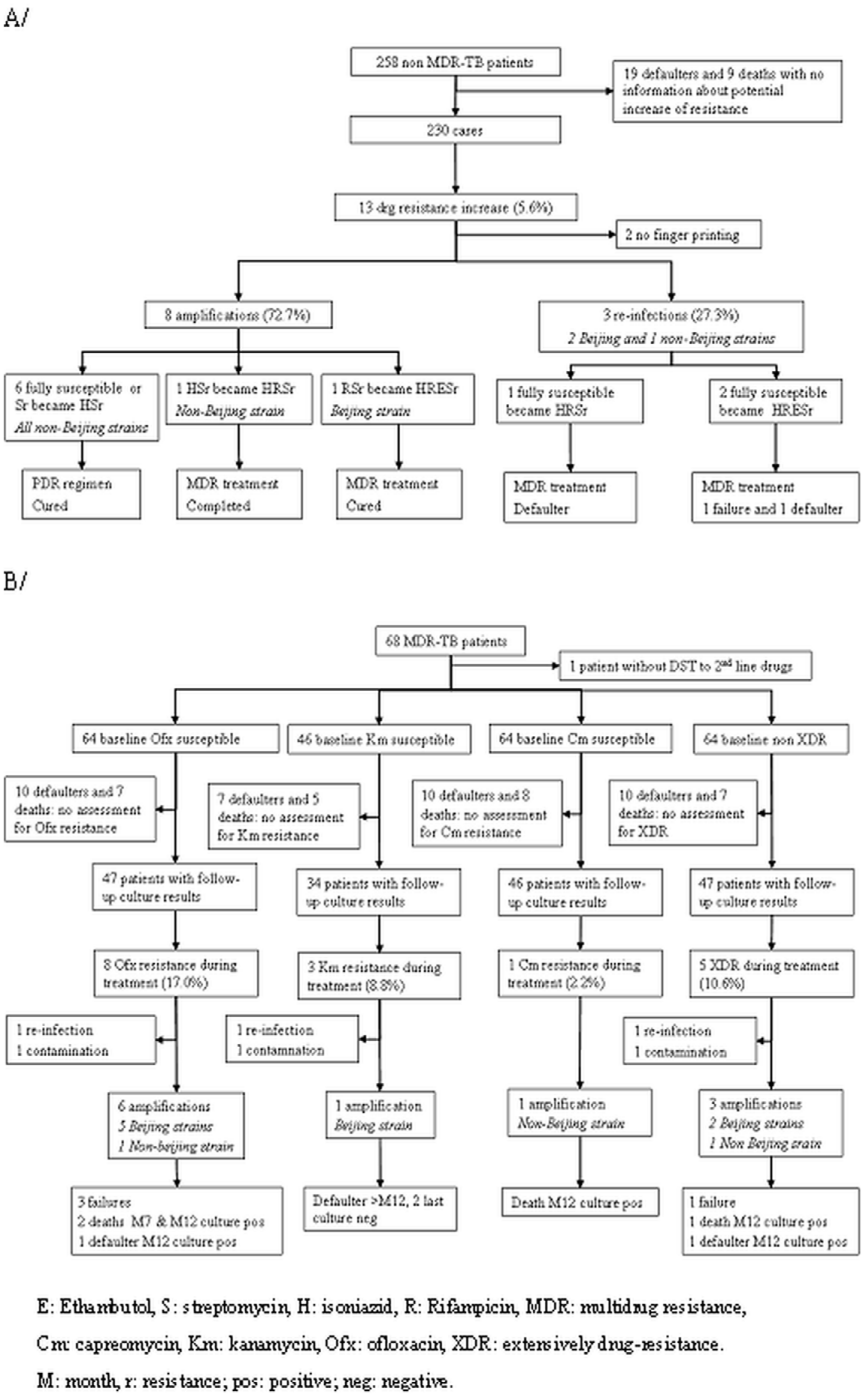
Evolution of drug resistance during treatment: amplification and re-infection. A. Drug resistance increase in fully susceptible and PDR-TB patients during treatment B. Drug resistance increase to ofloxacin, kanamycin, capreomycin and XDR during MDR-TB treatment.

Figure 1 shows patterns of drug resistance amplification and re-infection during treatment. Three patients with fully susceptible *MTB* strains were re-infected with a MDR-TB strain and had negative outcomes despite treatment adaptation. Cluster analysis showed that for one of them, the index case was a fellow patient hospitalised during the same period. This cluster included the strains from these 2 patients. Another patient was infected one month after starting treatment with a strain belonging to a cluster of MDR-TB strains identified in 3 other patients. They were all hospitalised for MDR-TB treatment during the same period as the contact case. The last patient was infected with a strain belonging to a cluster of MDR-TB strains identified in 15 patients. Three patients were hospitalised for MDR-TB treatment when the contact was regularly visiting the hospital to receive ambulatory treatment.

Among 58 PDR-TB strains of patients with complete follow-up culture and finger printing results, 2 amplified to MDR-TB during treatment (3.4%). 1/7 (14.3%) and 1/48 (2.1%) PDR-TB patients were infected with a Beijing strains and with a non-Beijing strain who amplified to MDR-TB, respectively (Fig. 1A).

Of the 47 MDR-TB patients susceptible to Ofx at baseline with follow-up culture results, 8 developed Ofx resistance (Fig. 1B). DNA fingerprinting was possible for 7 of these (1 culture was contaminated) showing re-infection with a XDR-TB strain in one patient and amplification to Ofx in 6 patients. The amplification rate to Ofx was 13.3% (6/45). It was 3.1% (1/32) and 2.2% (1/46) for Km and Cm, respectively. The Ofx resistance amplification rate was higher in MDR-TB patients infected with a Beijing strain 5/29 (17.2%) compared with those infected with a non-Beijing strain 1/16 (6.2%). However, this does not reach statistical significance (p = 0.56). Finally, of the 46 MDR-TB patients who were not XDR-TB at baseline and had valid follow-up culture results and RFLP results, 3 amplified to XDR TB during treatment (6.7%) and one was re-infected with a Beijing XDR-TB strain. The increase in Ofx resistance during treatment was significantly associated with a negative outcome (p<0.001).

## Discussion

Our study demonstrates that in a geographic area with high MDR-TB prevalence, such as Abkhazia, a treatment strategy based on early detection of drug resistance and treatment adaptation can be highly successful in patients with fully drug susceptible, mono- and poly-drug resistant strains but not in MDR-TB patients. Less than 1% (2/228) of these patients amplified resistance to MDR-TB on TB treatment. However, three patients with fully susceptible strains were re-infected with an MDR-TB strain, most likely in the TB hospital, despite infection control measures being in place, including the separation between susceptible, PDR and MDR-TB patients. Due to the absence of HIV testing in this study, it was not possible to assess if the nosocomial transmission could be explained by a high rate of TB-HIV infection. However, this seems unlikely given the low HIV prevalence among adults in Georgia (0.1% in 2007) [22]. The centralisation of drug resistance treatment and the long duration of hospitalisation of MDR-TB patients might have increased the risk of nosocomial transmissions [23], [24].

Contrary to the situation of patient with susceptible and PDR-TB, the TB control strategy in place in Abkhazia failed to successfully treat MDR-TB patients, despite the use of individualized treatment regimens in line with international recommendations. Indeed, the success rate (32%) was substantially lower than the average success rate of 64% (28–87 range) reported in the last published meta-analysis of 29 studies using individualized treatment regimens [4]. Our data are more in accordance with unpublished results from a recent meta-analysis, reporting an average success rate of 54%, and below 50% in several high incidence settings [25]. This last meta-analysis includes data from published and unpublished cohorts, which can reduce the risk of publication bias and could explain the lower success rate compared with previous meta-analysis. One factor contributing to the low MDR-TB treatment success rate in Abkhazia is the high death rate (23%) [4]. Most of the deaths occurred very early after starting treatment and could be due to late diagnosis. Since the TB drug resistance program in Abkhazia started in 2001 and was still new at the beginning of the study (2003), the backlog of undiagnosed MDR-TB patients could explain why some patients were diagnosed late in an advanced stage of the disease. The HIV-TB co-infection rate was estimated to be low in the region and is unlikely to contribute significantly to the high death rate observed in our study.

The high defaulter rate also contributes to the poor treatment success of MDR-TB patients in Abkhazia. Combinations of several factors resulting from the difficult social and economical conditions (chronic conflict and 10 years of economic blockade) are likely to contribute to this problem. Despite big efforts, good hospitalisation conditions, social support, and early management of side effects, patients had difficulties in tolerating long hospitalisation. Earlier discharge from hospital and decentralised treatment, which started in 2007, may enhance treatment effectiveness through better adherence.

Due to the small sample size of MDR-TB patients, we couldn't confirm the association between baseline resistance to Ofx and negative outcomes in our study, as it had been shown in previous studies [26], [27]. The only predictor of poor outcomes in our study was the increase to Ofx resistance during treatment. Indeed, 17% of MDR-TB patients became resistant to Ofx. Resistance amplification could be confirmed in two third of them. Amplification to Ofx resistance during MDR-TB treatment has already been reported, and it can seriously jeopardise the patient's chance to be cured [10], [28], [29]. Interestingly, resistance amplification was nearly completely restricted to Beijing genotype strains, although this association was not statistically significant probably due to the small numbers. In line with previous reports it might be speculated that Beijing genotype strains have a higher capacity to develop drug resistances in case of pre-existing resistance, resulting in a selective advantage for Beijing genotype strains in areas with higher levels of drug resistance [7]. This is in accordance with the high rates of Beijing genotype strains found in several regions of the former Soviet Union and the observed association with MDR-TB resistance. The factors that lead to resistance amplification under adequate treatment regimens need to be urgently identified, in order to avoid ongoing XDR-TB development in high incidence settings.

This prospective cohort gave the opportunity to evaluate the recently established WHO case definitions for MDR-TB culture conversion [12]. Four out of 37 patients (11%) who converted their culture according to the WHO definition reverted to positive. The relevance of using two consecutive negative culture results as a definition for culture conversion to determine treatment duration should be further examined [12], [30].

In conclusion, in Abkhazia, the treatment strategy was effective for susceptible TB and for mono- and poly-drug resistant TB but not for MDR-TB. Ensuring adherence to long and poorly effective treatment regimens, especially for patients living in difficult socio-economical conditions, is a real challenge in Abkhazia. These outcomes and particularly the amplification from MDR- to XDR-TB plead for the development of more effective and shorter regimens. Large multicentric cohort analyses are required to further investigate optimal treatment regimens for MDR-TB with existing drugs. The evidence of MDR-TB nosocomial transmission emphasizes the paramount importance of infection control in hospitals and of more decentralized and outpatients approaches for treating drug resistant TB. In high MDR burden and limited resource countries, the programmatic impact of using the new molecular methods (eg. XpertMTB/RIF® test), which does not require heavy laboratory infrastructure, should be assessed. Indeed, these methods would allow faster identification of rifampicin resistance in both smear-positive and smear-negative patients, and a more rapid initiation of empirical MDR regimen, while waiting for full DST results. It is likely that earlier MDR treatment would reduce the case fatality rate and the risk of nosocomial transmission. XpertMTB/RIF® was recently introduced in the Abkhazia TB program.
